# Supplementary material for: PPARα-Selective Antagonist GW6471 Inhibits Cell Growth in Breast Cancer Stem Cells Inducing Energy Imbalance and Metabolic Stress
Source: Biomedicines. 2021 Jan 28;9(2):127. doi: 10.3390/biomedicines9020127 (PMC7912302; doi:10.3390/biomedicines9020127)
Supplement: Supplementary file 1 [file biomedicines-09-00127-s001.zip › biomedicines-1086999-proofreading-supp/Supplementary Table S1.docx]

**FIG 1B CYTOTOXICITY**

| Bonferroni's multiple comparisons test | Mean Diff, | 95,00% CI of diff, | Summary | Adjusted P Value |
| --- | --- | --- | --- | --- |
|  |  |  |  |  |
| CTR - GW6471  Time (h) |  |  |  |  |
| 0 | -2,699 | -10,19 to 4,789 | ns | >0,9999 |
| 6 | -3,181 | -10,67 to 4,307 | ns | >0,9999 |
| 12 | -3,764 | -11,25 to 3,724 | ns | >0,9999 |
| 18 | -4,236 | -11,72 to 3,252 | ns | >0,9999 |
| 24 | -5,793 | -13,28 to 1,695 | ns | 0,3218 |
| 30 | -6,755 | -14,24 to 0,7333 | ns | 0,1169 |
| 36 | -8,695 | -16,18 to -1,207 | * | 0,0107 |
| 42 | -12,65 | -20,14 to -5,162 | *** | <0,0001 |
| 48 | -15,76 | -23,25 to -8,271 | *** | <0,0001 |
| 54 | -20,12 | -27,61 to -12,64 | *** | <0,0001 |
| 60 | -24,06 | -31,55 to -16,57 | *** | <0,0001 |
| 66 | -26,36 | -33,85 to -18,88 | *** | <0,0001 |
| 72 | -28,64 | -36,12 to -21,15 | *** | <0,0001 |

**FIG 1C WHOLE SPHEROID BF**

| Bonferroni's multiple comparisons test | Mean Diff, | 95,00% CI of diff, | Summary | Adjusted P Value |
| --- | --- | --- | --- | --- |
|  |  |  |  |  |
| CTR - GW6471  Time (h) |  |  |  |  |
| 0 | 2,331 | -12,88 to 17,55 | ns | >0,9999 |
| 6 | 4,078 | -11,14 to 19,29 | ns | >0,9999 |
| 12 | 3,759 | -11,46 to 18,97 | ns | >0,9999 |
| 18 | 7,130 | -8,085 to 22,35 | ns | >0,9999 |
| 24 | 5,269 | -9,946 to 20,48 | ns | >0,9999 |
| 30 | 6,987 | -8,228 to 22,20 | ns | >0,9999 |
| 36 | 9,465 | -5,750 to 24,68 | ns | 0,9153 |
| 42 | 12,06 | -3,154 to 27,28 | ns | 0,2790 |
| 48 | 15,58 | 0,3602 to 30,79 | * | 0,0403 |
| 54 | 15,62 | 0,4054 to 30,84 | * | 0,0392 |
| 60 | 18,41 | 3,200 to 33,63 | * | 0,0065 |
| 66 | 21,54 | 6,322 to 36,75 | ** | 0,0007 |
| 72 | 25,06 | 9,848 to 40,28 | *** | <0,0001 |

**FIG 2D CASPASE-3/7**

| Bonferroni's multiple comparisons test | Mean Diff, | 95,00% CI of diff, | Summary | Adjusted P Value |
| --- | --- | --- | --- | --- |
|  |  |  |  |  |
| CTR - GW6471  Time (h) |  |  |  |  |
| 0 | -0,9783 | -1,537 to -0,4196 | *** | <0,0001 |
| 6 | -1,480 | -2,039 to -0,9214 | *** | <0,0001 |
| 12 | -1,480 | -2,039 to -0,9216 | *** | <0,0001 |
| 18 | -2,009 | -2,568 to -1,450 | *** | <0,0001 |
| 24 | -2,110 | -2,668 to -1,551 | *** | <0,0001 |
| 30 | -2,520 | -3,079 to -1,961 | *** | <0,0001 |
| 36 | -2,801 | -3,360 to -2,242 | *** | <0,0001 |
| 42 | -3,206 | -3,765 to -2,648 | *** | <0,0001 |
| 48 | -3,781 | -4,340 to -3,223 | *** | <0,0001 |
| 54 | -4,732 | -5,290 to -4,173 | *** | <0,0001 |
| 60 | -5,586 | -6,145 to -5,028 | *** | <0,0001 |
| 66 | -6,822 | -7,380 to -6,263 | *** | <0,0001 |
| 72 | -8,920 | -9,479 to -8,362 | *** | <0,0001 |

**FIG 7C INVADING CELLS AREA BF**

| Bonferroni's multiple comparisons test | Mean Diff, | 95,00% CI of diff, | Summary | Adjusted P Value |
| --- | --- | --- | --- | --- |
|  |  |  |  |  |
| CTR - GW6471  Time (h) |  |  |  |  |
| 0 | -0,06500 | -0,4419 to 0,3119 | ns | >0,9999 |
| 6 | 0,03100 | -0,3459 to 0,4079 | ns | >0,9999 |
| 12 | 0,2073 | -0,1695 to 0,5842 | ns | >0,9999 |
| 18 | 0,6578 | 0,2809 to 1,035 | *** | <0,0001 |
| 24 | 0,3535 | -0,02332 to 0,7304 | ns | 0,0862 |
| 30 | 0,6132 | 0,2363 to 0,9901 | *** | <0,0001 |
| 36 | 0,7756 | 0,3987 to 1,152 | *** | <0,0001 |
| 42 | 0,5758 | 0,1989 to 0,9526 | *** | 0,0002 |
| 48 | 0,9941 | 0,6172 to 1,371 | *** | <0,0001 |
| 54 | 0,8941 | 0,5172 to 1,271 | *** | <0,0001 |
| 60 | 1,186 | 0,8089 to 1,563 | *** | <0,0001 |
| 66 | 0,7790 | 0,4021 to 1,156 | *** | <0,0001 |
| 72 | 1,235 | 0,8580 to 1,612 | *** | <0,0001 |
