# Supplementary figures and images for: PPARα-Selective Antagonist GW6471 Inhibits Cell Growth in Breast Cancer Stem Cells Inducing Energy Imbalance and Metabolic Stress
Source: Biomedicines. 2021 Jan 28;9(2):127. doi: 10.3390/biomedicines9020127 (PMC7912302; doi:10.3390/biomedicines9020127)

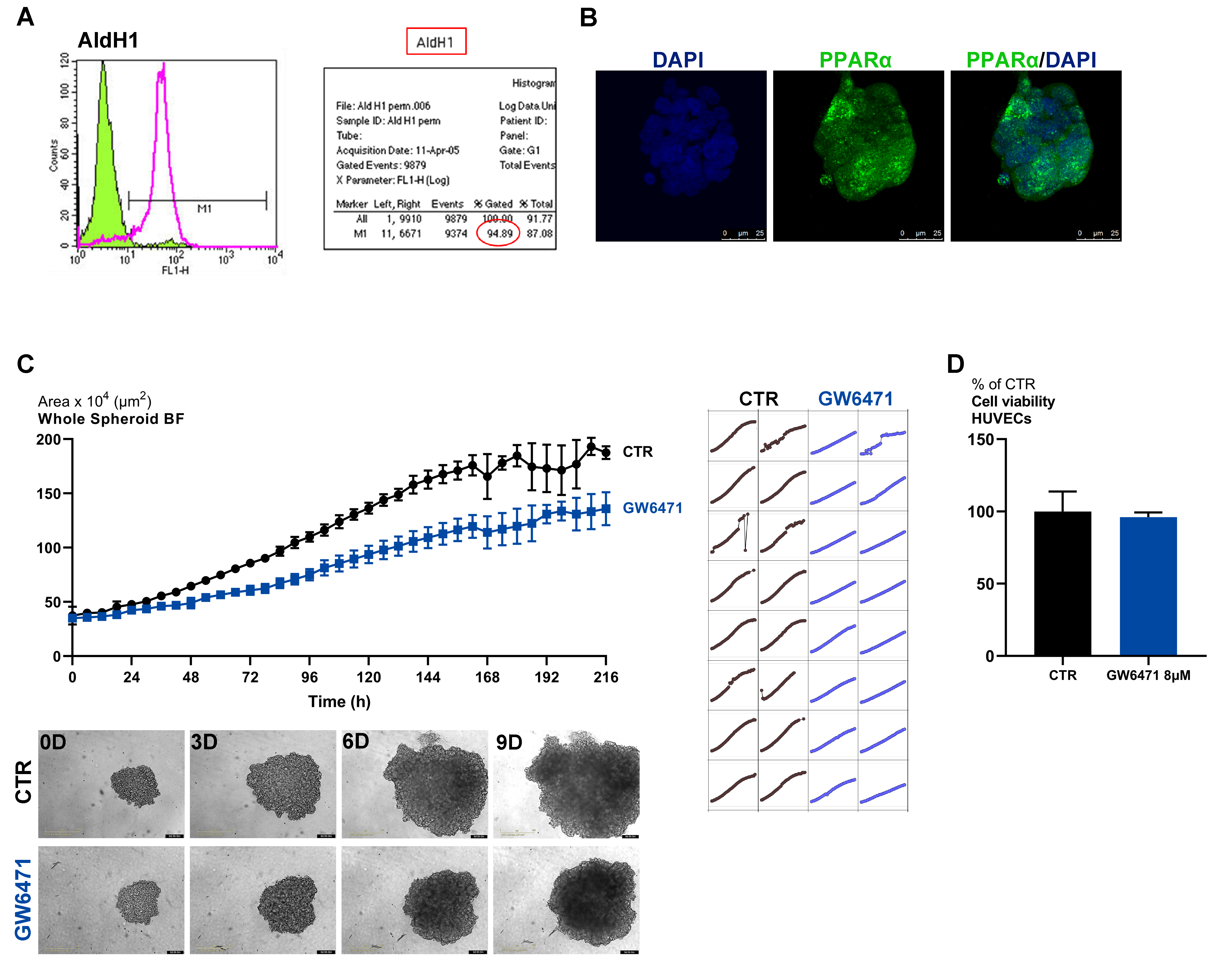

Supplement: Supplementary file 1 [file biomedicines-09-00127-s001.zip › biomedicines-1086999-proofreading-supp/Supplementary Figure S1.tif]
